# Antioxidant and Anti-Inflammatory Activities of Berberine in the Treatment of Diabetes Mellitus

**DOI:** 10.1155/2014/289264

**Published:** 2014-02-11

**Authors:** Zheng Li, Ya-Na Geng, Jian-Dong Jiang, Wei-Jia Kong

**Affiliations:** ^1^Department of Pharmacology, Institute of Medicinal Biotechnology, Chinese Academy of Medical Sciences and Peking Union Medical College, Beijing 100050, China; ^2^State Key Laboratory of Bioactive Natural Products and Function, Institute of Materia Medica, Chinese Academy of Medical Sciences and Peking Union Medical College, Beijing 100050, China

## Abstract

Oxidative stress and inflammation are proved to be critical for the pathogenesis of diabetes mellitus. Berberine (BBR) is a natural compound isolated from plants such as *Coptis chinensis* and *Hydrastis canadensis* and with multiple pharmacological activities. Recent studies showed that BBR had antioxidant and anti-inflammatory activities, which contributed in part to its efficacy against diabetes mellitus. In this review, we summarized the antioxidant and anti-inflammatory activities of BBR as well as their molecular basis. The antioxidant and anti-inflammatory activities of BBR were noted with changes in oxidative stress markers, antioxidant enzymes, and proinflammatory cytokines after BBR administration in diabetic animals. BBR inhibited oxidative stress and inflammation in a variety of tissues including liver, adipose tissue, kidney and pancreas. Mechanisms of the antioxidant and anti-inflammatory activities of BBR were complex, which involved multiple cellular kinases and signaling pathways, such as AMP-activated protein kinase (AMPK), mitogen-activated protein kinases (MAPKs), nuclear factor erythroid-2-related factor-2 (Nrf2) pathway, and nuclear factor-**κ**B (NF-**κ**B) pathway. Detailed mechanisms and pathways for the antioxidant and anti-inflammatory activities of BBR still need further investigation. Clarification of these issues could help to understand the pharmacology of BBR in the treatment of diabetes mellitus and promote the development of antidiabetic natural products.

## 1. Introduction

For decades, diabetes mellitus especially type 2 diabetes mellitus (T2DM) has become a public health problem and threatened the people worldwide, not only in western countries, but also in the developing world like China. The pathogenesis and pathophysiological processes of T2DM are extreme complex and remain to be controversial. However, a growing number of evidences showed that oxidative stress and inflammation might play important roles in the development of T2DM [1, 2].

In metabolic disorders, oxidative stress could be typically induced by excessive nutritional factors like glucose and free fatty acids (FFA) [3, 4]. Oxidative stress could induce or deteriorate insulin resistance and diabetes through multiple mechanisms. In the process of oxidative stress, excessive reactive oxygen species (ROS) were produced, mainly by mitochondria [5]. ROS include superoxide anion (O_2_^−•^), hydroxyl radical (OH^•^), hydrogen peroxide (H_2_O_2_) [6]. They could cause damage and apoptosis of pancreatic islet *β*-cells and reduction of insulin secretion [7]. ROS could activate cellular signaling pathways like c-jun N-terminal kinase (JNK), protein kinase C (PKC), and nuclear factor-*κ*B (NF-*κ*B) and then interfere with the insulin signaling pathway and cause insulin resistance [8–10]. In addition, oxidative stress also contributes to the development of chronic complications of diabetes, such as diabetic nephropathy, retinopathy, and neuropathy [6].

Recently, results from laboratory studies as well as clinical investigations have proved that diabetes is in fact an inflammatory disease [2, 11]. The importance of inflammation in insulin resistance, diabetes and complications has been critically reviewed [12–16], concerning relevant proinflammatory cytokines as well as cellular signaling pathways. In the pathogenesis of diabetes mellitus, proinflammatory cytokines such as tumor necrosis factor-*α* (TNF-*α*) and interleukin-6 (IL-6) could be produced by immunocytes (like macrophages) and adipocytes [12–14]. TNF-*α* and IL-6 were important mediators of insulin resistance, as they could induce serine phosphorylation of insulin receptor substrate (IRS) through activation of JNK or NF-*κ*B pathway [14, 16]. Furthermore, overproduction of TNF-*α* and IL-6 in pancreas could cause islet dysfunction and accelerate the progression of diabetes [13].

There are a number of medications available in clinic to treat T2DM. In addition to chemical drugs, a number of natural products or traditional Chinese medicine (TCM) formulas were reported to have antidiabetic or insulin-sensitizing activities, and some of them have been used in clinic with a long history [11, 17, 18]. Berberine (BBR) is a natural compound isolated from plants such as *Coptis chinensis* and *Hydrastis canadensis* and with multiple pharmacological activities [19]. The emerging role of BBR in modifying sugar and lipid metabolism has been verified in a large amount of experimental and clinical studies [20–23].

In general, BBR was safe and effective in the treatment of patients with T2DM [19–21, 23, 24]. The glucose-lowering efficacy of BBR was close to those of metformin and rosiglitazone [23, 24]. Notably, due to the low toxicity, BBR could be used in diabetic patients with chronic hepatitis [24]. Beneficial effects of BBR were also observed in combating diabetic complications. Diabetes related endothelial dysfunction, nephropathy, and neuropathy could be relieved after BBR administration [21].

Mechanisms of BBR in restoring insulin sensitivity and lowering blood glucose included inhibition of mitochondrial function and activation of AMP-activated protein kinase (AMPK), regulation of islet function, modulation of gut microenvironment, and upregulation of insulin receptor expression [20–22, 24–26]. Recent studies showed that BBR had beneficial effects on cellular oxidative stress and inflammation, which could also play important roles in its activity against diabetes mellitus. In this paper, we reviewed the antioxidant and anti-inflammatory activities as well as their molecular basis of BBR in treating diabetes mellitus and insulin resistance.

## 2. Antioxidant Activity and Mechanisms of BBR in Treating Diabetes Mellitus

### 2.1. BBR Reduced Oxidative Stress in Diabetes Mellitus

The inhibitory effect of BBR on oxidative stress was observed both in cells cultured with high glucose-containing media [38] and in a series of diabetic animal models (Table 1) [27–30, 32–37]. The antioxidant activity of BBR was revealed by changes of oxidative stress markers as well as antioxidant enzymes. Oxidative stress markers include malondialdehyde (MDA), a product of lipid peroxidation which increases during oxidative stress [39], as well as glutathione (GSH), which often declines during oxidative stress [40]. GSH is an antioxidant itself and is a substrate of glutathione peroxidase (GSH-Px) in the clearance of peroxides [40]. In addition to GSH-Px, another well-known antioxidant enzyme, superoxide dismutase (SOD), is also involved to evaluate the inhibitory effect of BBR on oxidative stress. Antioxidant enzyme is a part of the antioxidant defense mechanisms, which helps to maintain the balance of redox in organisms and could be damaged in the pathogenesis of diabetes mellitus [41].

As summarized in Table 1, the majority of reports [27–30, 32–37] supported the antioxidant activity of BBR in diabetic animal models, which were induced by streptozotocin (STZ) or alloxan injection with or without high fat diet (HFD) feeding. The relief of oxidative stress by BBR was noted by changes of oxidative stress markers as well as antioxidant enzymes. In general, BBR administration decreased MDA content and increased the contents of SOD, GSH and GSH-Px, which would help to scavenge excessive free radicals and overcome oxidative stress [40, 41]. In only one of the reports [31], the effect of BBR on oxidative stress seemed not to be obvious. However, in this report [31], the contents of MDA and SOD of the diabetic animals did not have statistically significant difference as compared with those of the normal control animals, inconsistent with the results from other reports [27–30, 32–37]. In another report [30], BBR treatment greatly upregulated the mRNA level of SOD but decreased GSH and GSH-Px contents in diabetic mice. In this report [30], while SOD was observed to be reduced in diabetic mice, the amounts of GSH and GSH-Px were increased, probably due to the acute stage of experimental diabetes mellitus [52], which usually occurred within 3-4 weeks of STZ injection [53, 54]. So, it seems that BBR could regulate the GSH/GSH-Px antioxidant mechanism differentially at different stages of diabetes mellitus.

The antioxidant activity of BBR was associated with its inhibitory effect on the development of diabetes mellitus and insulin resistance induced by STZ/alloxan + HFD in animals [32–35]. Furthermore, BBR inhibited oxidative stress in a variety of tissues, such as the serum [27–29], liver [29, 30, 32], kidney [32, 33], pancreas [34], heart [35] and central nerve system [36, 37]. The inhibitory effect of BBR on oxidative stress was associated with its activity against renal injury [27, 28, 33], pancreatic islet dysfunction [34], and memory impairment [36] in diabetic animals.

### 2.2. Mechanisms and Pathways of BBR against Oxidative Stress

**Figure 1:**
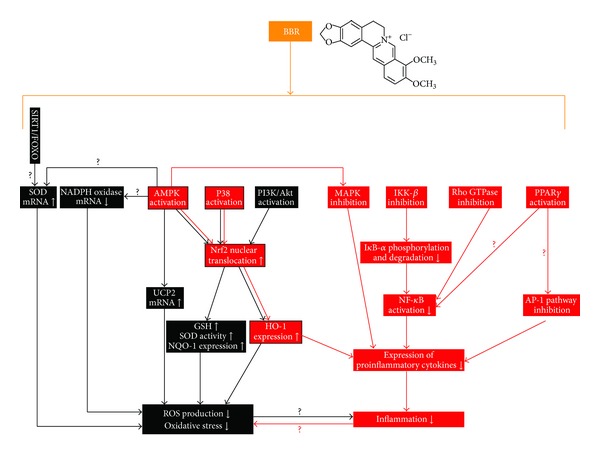
Schematic illustration of the molecular mechanisms and pathways of BBR in reducing oxidative stress and inflammation. (1) BBR could inhibit oxidative stress by upregulation of SOD, UCP2 and downregulation of NADPH oxidase expression, which was possible to be mediated by the SIRT1/FOXO or AMPK pathway. (2) BBR administration induced the activation of the Nrf2 pathway, which was crucial for the antioxidant and anti-inflammatory activities of BBR. The effect of BBR on Nrf2 relied on the activation of AMPK, PI3K/Akt, and P38 pathways. (3) BBR could suppress inflammation by blocking the MAPK pathways in an AMPK-dependent manner, inhibiting the classic NF-*κ*B signaling pathway; inhibiting the Rho GTPase pathway, which was proved to play a role in NF-*κ*B regulation, and attenuating the transcription activity of AP-1, which was possible to be mediated by PPAR*γ* activation. The black lines/boxes and red lines/boxes indicate pathways and molecules involved in the antioxidant or anti-inflammatory activity of BBR, respectively. Red boxes with black frame indicate pathways and molecules responsible for both antioxidant and anti-inflammatory activities of BBR. The symbols of “?” indicate possible mechanisms and pathways that need to be further verified. There was a vicious cycle between oxidative stress and inflammation, which was possibly able to be terminated by BBR administration.

Molecular mechanisms of BBR in reducing oxidative stress seem to be related with multiple cellular pathways and need further investigation. The schematic illustration of the pathways from available data was presented in Figure 1.

It was reported that BBR scavenged superoxide free radicals directly in vitro in a system containing alkaline dimethyl sulfoxide (DMSO) [55]. The mRNA expression level of SOD could be upregulated by BBR in diabetic mice, and it played a role in BBR's activity against oxidative stress [30, 32]. Also, BBR was reported to increase the expression level of sirtuin 1 (SIRT1) [56], a deacetylase with multiple biological activities and antioxidant activity [57]. In oxidative stress, SIRT1 could induce deacetylation of the forkhead box O (FOXO) transcription factors and increased the transcription of their target genes, which included *SOD* [58]. It is possible that BBR increased SOD expression *via* the SIRT1/FOXO pathway, more experimental evidences are needed to support this point.

BBR could reduce oxidative stress by attenuating the expression level of nicotinamide adenine dinucleotide phosphate (NADPH) oxidase, which was a major source of ROS production in cells [59, 60]. NADPH oxidase was able to be upregulated by high levels of fatty acids, glucose or advanced glycation end products (AGEs), resulting in overproduction of ROS [3, 4, 10]. Among various NADPH oxidase isoforms, BBR was reported to suppress the overexpression of NADPH oxidase 2/4 and decrease ROS production in macrophages and endothelial cells upon stimulation with inflammatory stimuli [59, 60].

Activation of NADPH oxidase is associated with the onset of diabetes, obesity and arthrosclerosis [3, 4, 10]. Now, NADPH oxidase is considered to be a potential target to treat diabetes and related complications [61, 62]. The inhibitory effect of BBR on NADPH oxidase and the subsequent ROS reduction could partially explain its beneficial effects on diabetic complications, such as nephropathy and endothelial dysfunction [21, 60]. NADPH oxidase could be negatively regulated by AMP-activated protein kinase (AMPK) activation [63, 64], which was involved in the activities of BBR against diabetes mellitus [20]. However, whether BBR downregulated NADPH oxidase through AMPK activation, or not, remains unclear and need direct evidence.

AMPK seemed to play a pivotal role in mediating the antioxidant activity of BBR. Besides NADPH oxidase downregulation, AMPK activation was linked to the upregulation of SOD expression [65, 66], which was observed in BBR treated diabetic mice [30, 32]. In addition, BBR could increase the expression of uncoupling protein 2 (UCP2) and inhibit oxidative stress in the artery of mice in an AMPK-dependent manner [67]. UCP2 is a member of the mitochondrial inner membrane proteins and was shown to be negatively related to ROS production and oxidative stress [68, 69]. Conflicting results existed concerning the effect of BBR on the expression of UCP2. For example, in another report [70], BBR decreased the expression level of UCP2 in the liver in a rat model of nonalcoholic fatty liver disease (NAFLD) and reduced hepatic steatosis. Whether or not BBR regulates UCP2 in a tissue-specific manner is unknown and needs more study.

The relationship between UCP2 and diabetes mellitus was complex. On the one hand, upregulation of UCP2 in adipose tissue or kidney could reduce ROS production and relieve diabetes or relevant complication; but on the other hand, increased UCP2 in islet *β*-cells was related to reduction of insulin secretion [71]. How UCP2 is regulated by BBR in *β*-cells is unknown and needs investigation.

Recent studies revealed that BBR suppressed oxidative stress through induction of the nuclear factor erythroid-2-related factor-2 (Nrf2) pathway [72–75]. Nrf2 was an antioxidant transcription factor mediating the expression of antioxidant enzymes like NADPH quinine oxidoreductase-1 (NQO-1) and heme oxygenase-1 (HO-1) [76]; it had a wide range of activities in regulating redox state and energy metabolism in cells [76]. Now, Nrf2 is recognized as an important mediator of BBR in reducing oxidative stress, as blocking Nrf2 abolishes the antioxidant activity of BBR in macrophages and nerve cells [72–75]. The activity of BBR on Nrf2 relied on the activation of several cellular signaling pathways including the AMPK pathway, phosphatidylinositol 3-kinase (PI3K)/Akt pathway, and the P38 pathway (Figure 1), as blocking these pathways could diminish the stimulating effect of BBR on Nrf2 [72–75]. BBR activated these pathways, and then induced nuclear translocation of Nrf2 which could activate the expression of antioxidant enzymes, increase SOD and GSH contents in cells and reduce ROS production and oxidative stress (Figure 1) [72–75].

## 3. Anti-Inflammatory Activity and Mechanisms of BBR in the Treatment of Diabetes Mellitus

### 3.1. BBR Reduced Inflammation Response in Diabetes Mellitus

The anti-inflammatory activity of BBR was observed both in vitro and in vivo and was noted by the reduction of proinflammatory cytokines as well as acute phase proteins (Table 2) [28, 42–51]. In cultured metabolic cells (adipocytes and liver cells), immunocytes (macrophages and splenocytes) or pancreatic *β*-cells, BBR treatment reduced the production of TNF-*α*, IL-6, IL-1*β*, matrix metalloprotease 9 (MMP9), cyclooxygenase-2 (COX2), inducible nitric oxide synthase (iNOS), monocyte chemoattractant protein 1 (MCP-1), and C-reaction protein (CRP) and haptoglobin (HP) [42–46]. In insulin resistant HepG2 cells [43], the anti-inflammatory activity of BBR was associated with its insulin-sensitizing effect. BBR administration significantly decreased cytokine production, and reduced serine phosphorylation but increased insulin-mediated tyrosine phosphorylation of IRS in HepG2 cells treated with palmitate [43].

BBR could reduce proinflammatory cytokines, acute phase protein and infiltration of inflammatory cells in animals with diabetes mellitus or insulin resistance, either induced by STZ injection/HFD feeding or spontaneously happened (Table 2) [28, 44, 47–49]. In these animal models, the anti-inflammatory activity of BBR was observed in different tissues like serum, liver, adipose tissue, and kidney and was associated with its effect against insulin resistance or diabetes mellitus [28, 44, 47–49].

Notably, BBR was proved to inhibit inflammation and relieve the development of type 1 diabetes mellitus in NOD mice [50, 51]. As shown in Table 2, BBR reduced the production of proinflammatory cytokines like TNF-*α*, IL-6, interferon-*γ* (IFN*γ*), and IL-17 in NOD mice [50, 51]. Furthermore, BBR could increase the ratios of anti-inflammatory/proinflammatory cytokines, like IL-10/IL-1*β*, IL-10/IL-6 and IL-10/TNF-*α* [51]. The anti-inflammatory activity of BBR was observed in splenocytes, kidney, and liver of NOD mice [50, 51].

Besides evidences from cultured cells and diabetic animal models, the anti-inflammatory effect of BBR was also observed in clinic [77]. BBR therapy at a dose of 1 g/day for 3 months significantly reduced the serum IL-6 level in patients with T2DM [77].

### 3.2. Mechanisms and Pathways of the Anti-Inflammatory Activity of BBR

BBR suppresses inflammation through complex mechanisms. Representative cellular pathways of BBR in inhibiting inflammation, which apparently shared in part with the antioxidant pathways, are summarized in Figure 1.

In addition to antioxidant activity, the AMPK pathway was also crucial for the anti-inflammatory efficacy of BBR [44, 72]. Blocking AMPK could abolish the inhibitory effect of BBR on the production of proinflammatory cytokines, like iNOS and COX2 in macrophages (Figure 1) [44, 72]. Excessive iNOS in cells could cause overproduction of nitric oxide and had close relationship with the development of insulin resistance [78]. COX2 was a key enzyme for the synthesis of prostaglandins [79], which were important mediators for the pathogenesis of diabetes mellitus and diabetic nephropathy [80].

The anti-inflammatory activity of BBR was also associated with its inhibitory effect on the mitogen-activated protein kinase (MAPK) signaling pathways, which were activated by inflammatory stimuli [44, 46, 81, 82]. The inhibitory effect of BBR on MAPKs was dependent on AMPK activation in macrophages [44]. It seems that conflicting results exist concerning the regulatory effect of BBR on MAPK signaling. Although some results suggested that BBR suppressed inflammation through inhibiting MAPKs [44, 46, 81, 82], others indicated that P38 was activated by BBR which was considered important for BBR's efficacy against oxidative stress and inflammation [75, 83]. In reviewing published results, it seemed that BBR could increase P38 phosphorylation in unstimulated cells with baseline P38 activity [83], but decrease MAPK phosphorylation in cells treated with inflammatory stimuli like lipopolysaccharide (LPS), FFA and TNF-*α* which could activate MAPKs [44, 46, 81, 82].

There was discrepancy concerning the role of P38 in BBR-stimulated glucose metabolism as well. For example, BBR was shown to activate P38 and increase glucose uptake in L6 cells; and the effect of BBR on glucose metabolism could be partially blocked by a P38 inhibitor [84]. But in another report, the glucose uptake-stimulating effect of BBR was independent of P38 in adipocytes [85]. These results may suggest that as members of a complex signaling network, MAPKs could be regulated by BBR differentially; and their relationships to other signaling molecules and roles in the pharmacological effect of BBR need further study.

Besides antioxidant activity, the transcription factor of Nrf2 also played an important role in the anti-inflammatory activity of BBR (Figure 1) [72, 83]. Blocking Nrf2 abolished the inhibitory effect of BBR on the production of proinflammatory cytokines in macrophages [72, 83]. BBR treatment could induce the activation of AMPK and P38, which in turn promote nuclear translocation of Nrf2, and inhibit the production of proinflammatory cytokines [72, 83].

HO-1, an antioxidant enzyme whose expression was driven by Nrf2 [76], could be induced by BBR [72–75, 83]. Besides antioxidant activity [72–75], HO-1 was also involved in the anti-inflammatory activity of BBR, as blocking HO-1 with inhibitor could attenuate the inhibitory effect of BBR on the production of proinflammatory cytokines [83]. Now, a growing number of evidences proved that HO-1 was a very important molecule with integrated beneficial effects against insulin resistance, diabetes mellitus, oxidative stress and inflammation [86]. HO-1 could be a useful target for the development of novel anti-diabetic drugs in the future [87].

The NF-*κ*B pathway plays a key role in controlling inflammation [14]; it is a critical target for the anti-inflammatory activity of BBR, as well (Figure 1). In NF-*κ*B signaling pathway, I*κ*B kinase-*β* (IKK-*β*) could be activated by inflammatory stimuli like TNF-*α*, as well as nutritional factors like glucose and FFA [10, 14]. The activation of IKK-*β* required phosphorylation of the serine residue at position 181 (ser^181^) [88, 89]. In insulin resistant 3T3-L1 adipocytes [90] and liver/adipose tissues from obese mice feed with HFD [47], BBR administration greatly reduced phosphorylation of ser^181^ and activation of IKK-*β*. In addition, the inhibitory effect of BBR on IKK-*β* required a cysteine residue at position 179 of IKK-*β* [91].

As inhibitory *κ*B-*α* (I*κ*B-*α*) was phosphorylated by IKK-*β* and then degraded [88, 89], inhibition of IKK-*β* by BBR could result in the stabilization of I*κ*B-*α* [82, 92, 93], which in turn blocked the nuclear translocation of NF-*κ*B [46, 82, 90, 93, 94]. As a transcription factor, NF-*κ*B promoted the expression of various proinflammatory cytokines such as TNF-*α*, IL-6, iNOS and COX2 [10, 14]. In a variety of cells or tissues like pancreatic *β*-cells [46], nerve cells [82], lung cells [92], and rat kidney [93, 94] as well as in a mice model of insulin resistance [47], the inhibitory effect of BBR on the production of proinflammatory cytokines was related to its negative regulation of the NF-*κ*B signaling pathway.

Recent study proved that BBR could reduce renal inflammation in diabetic rats through inhibiting the Rho GTPase signaling pathway [28]. Rho GTPase was a member of the superfamily of small GTP binding proteins with multiple biological functions [95]; it was proved to positively regulate the NF-*κ*B signaling pathway in diabetic rats [96]. So, in addition to regulation of the classic NF-*κ*B signaling pathway, BBR could inhibit NF-*κ*B by suppressing Rho GTPase [28, 96]. Furthermore, the inhibitory effect of BBR on Rho GTPase relied on its antioxidant activity [28].

In addition to NF-*κ*B, transcription factor activator protein 1 (AP-1) also played a role in the anti-inflammatory activity of BBR [97, 98]. Like NF-*κ*B, AP-1 was critical for the development of inflammation [99]. Administration of BBR to macrophages or epithelial cells greatly attenuated the DNA binding activity of AP-1 and reduced the production of cytokines like MCP-1 and COX2 [97, 98].

There were reports that the transcription stimulating activity of AP-1 and NF-*κ*B could be inhibited by activation of peroxisome proliferator-activated receptor *γ* (PPAR*γ*) [100–102]. BBR was reported to reduce the production of proinflammatory cytokines partially through PPAR*γ* activation in macrophages and intestine [103, 104]. It is possible that PPAR*γ* activation may contribute in part to the inhibition of AP-1 and NF-*κ*B by BBR (Figure 1); direct experimental evidences are needed to support this presumption. However, the effect of BBR on PPAR*γ* remains uncertain. Some reports showed that BBR treatment could activate PPAR*γ* [103, 104] or increase its expression in adipose tissues [105, 106], but others suggested that BBR downregulated the expression of PPAR*γ* in adipocytes [107, 108]. The explanation for the difference is still unavailable.

## 4. Discussion

As a herbal compound, BBR was first reported to have glucose-lowering efficacy in 1986 in diabetic animals [109]. Then, in 1988, BBR was found to reduce the blood glucose level in patients with T2DM [110]. The molecular pharmacology of BBR in treating diabetes mellitus and insulin resistance was intensively studied in recent years and was reviewed in detail elsewhere [20–23]. Here in the present review, we summarized the activity of BBR against diabetes mellitus from a different point of view: the inhibitory effects of BBR on oxidative stress and inflammation.

From the view of metabolic disorders, inflammation and oxidative stress closely relates each other [14, 111, 112]. NF-*κ*B is a master regulator of both inflammation reaction and oxidative stress [10, 14, 111, 112]. Oxidative stress could stimulate the production of proinflammatory cytokines such as TNF-*α* and IL-6 in the adipose tissue [3]. On the other hand, proinflammatory cytokines could also increase the amount of ROS in cells and promote oxidative stress [113]. It was obvious that a vicious cycle existed between oxidative stress and inflammation, which, in collaboration, could deteriorate insulin resistance [14, 111]. BBR could reduce oxidative stress and inflammation through some of the common cellular signaling pathways, such as the AMPK pathway and Nrf2/HO pathway [72, 83]. It was rational to infer that BBR administration could terminate the vicious cycle between oxidative stress and inflammation. The inhibitory effect of BBR on the oxidative-inflammatory loop needs to be further studied.

BBR suppressed oxidative stress and inflammation through multiple mechanisms. In addition to what was mentioned above, recent studies indicated that the anti-inflammatory activity of BBR was also associated with its beneficial effects in the gut [114–116]. Due to the possible low bioavailability [117], high concentration of BBR in the gut after oral administration could modulate the structure of gut microbiota, resulting in the enrichment of short-chain fatty acid (SCFA)-producing bacteria in the gut [114]. SCFA could improve intestinal barrier function and prevent inflammation by blocking exogenous antigen or endotoxin to enter into the blood [118]. This theory was supported by the observations that BBR could ameliorate intestinal barrier damage induced by TNF-*α* or LPS in cultured human colon monolayer or animal models [119–121].

On the other hand, while BBR seemed poorly absorbed in the gut, recent pharmacokinetic study [122] suggested that the concentrations of BBR (together with its metabolites) in organs (like the liver and kidney) were significantly higher than its blood concentration after oral administration in rats. This finding could partially explain the pharmacological activities of BBR in various tissues despite its low blood concentration [117, 122]. Our previous work showed that the metabolites of BBR could activate AMPK, as well [123]. It will be interesting to investigate if BBR metabolites have any antioxidant or anti-inflammatory activity against diabetes mellitus.

Some of the key issues of BBR in reducing oxidative stress and inflammation still need to be further studied. For example, conflicting results in the regulation of UCP2, MAPKs, and PPAR*γ* by BBR need to be clarified. The clinical outcome and significance of the antioxidant and anti-inflammatory activities of BBR in treating diabetes mellitus need to be investigated. In addition, recent studies showed that BBR had beneficial effects against endoplasmic reticulum (ER) stress in insulin resistance and islet *β*-cell dysfunction [124, 125]. ER stress, which could be induced by ROS and inflammation [126, 127], is a key factor for the pathogenesis of diabetes mellitus and has become an important therapeutic target in recent years [128]. Further investigation of the influence of BBR on ER stress and its relationship to oxidative stress/inflammation will help to clarify the pharmacology of BBR against diabetes mellitus and promote the research and development of antidiabetic natural products.

## 5. Conclusion

In summary, natural compound BBR has antioxidant and anti-inflammatory activities which might contribute in part to its therapeutic efficacies against diabetes mellitus and insulin resistance. Multiple cellular kinases as well as signaling pathways such as AMPK, MAPKs, Nrf2/HO pathway, and NF-*κ*B pathway (Figure 1) were verified to be pivotal for BBR in reducing oxidative stress and inflammation. Because of the increased interest in BBR's clinical uses in the past 10 years, the molecular details for the antioxidant and anti-inflammatory activities of BBR merit further investigation.

## Figures and Tables

**Table 1:** Effects of BBR on parameters of oxidative stress in animals with diabetes mellitus.

References	Diabetic animal model	Administration of BBR	Tissues examined	Effects of BBR			
				Oxidative stress markers		Antioxidant enzymes	
				MDA	GSH	SOD	GSH-Px
[27]	Wistar rats, STZ 60 mg/kg, single i.p. injection	200 mg/kg/d, p.o. for 12 weeks	Serum	↓	ND	↑	ND

[28]	SD rats, STZ 60 mg/kg, single tail vein injection	200 mg/kg/d, p.o. for 12 weeks	Serum	↓	ND	↑	ND

[29]	Wistar rats, STZ 35 mg/kg, single i.p. injection, HFD for 14 weeks after 2 weeks on diabetes	75, 150, and 300 mg/kg/d, p.o. for 16 weeks	Serum and liver	↓	↑	↑	↑

[30]	ddY mice, STZ 100 mg/kg, single i.p. injection	200 mg/kg/d, p.o. for 2 weeks	Liver	ND	↓	↑	↓

[31]	SD rats, HFD for 2 weeks, then STZ 35 mg/kg, single i.p. injection	50, 100, and 150 mg/kg/d, p.o. for 6 weeks	Liver	—	—	—	ND

[32]	ICR mice, nicotinamide 1000 mg/kg + STZ 100 mg/kg, single i.p. injection	100 mg/kg/d, p.o. for 2 weeks	Liver and kidney	↓	ND	↑	ND

[33]	SD rats, HFD for 4 weeks, then STZ 40 mg/kg, single i.p. injection, HFD for another 8 weeks	100 and 200 mg/kg/d, p.o. for 8 weeks	Kidney	↓	ND	↑	ND

[34]	Wistar rats, STZ 35 mg/kg, single i.p. injection, HFD for 14 weeks after 2 weeks on diabetes	75, 150 and 300 mg/kg/d, p.o. for 16 weeks	Pancreas	↓	ND	↑	ND

[35]	Wistar rats, alloxan 55 mg/kg, single tail vein injection, then on HFD	100 and 200 mg/kg/d, p.o. for 21 days	Heart	↓	ND	↑	↑

[36]	Wistar rats, STZ 60 mg/kg, single i.p. injection	25, 50, and 100 mg/kg/d, p.o. for 30 days	Cortex and hippocampus	↓	↑	ND	ND

[37]	Wistar rats, STZ 55 mg/kg, single i.p. injection	50 and 100 mg/kg/d, p.o. for 8 weeks	Hippocampus	↓	ND	↑	ND

↓: decrease, ↑: increase, ND: not determined, —: no effect; BBR: berberine, MDA: malondialdehyde, GSH: glutathione, SOD: superoxide dismutase, GSH-Px: glutathione peroxidase, STZ: streptozotocin, p.o.: per os/oral administration, i.p.: intraperitoneal, HFD: high fat diet.

**Table 2:** Effects of BBR on inflammatory cytokines and inflammation in cultured cells or animals with diabetes mellitus or insulin resistance.

References	Cell type, animal model	Administration of BBR	Samples examined	Effects of BBR
[42]	3T3-L1 adipocytes	10 *μ*M for 18 hours	3T3-L1 adipocytes	↓: TNF-*α*, IL-6, CRP, and HP mRNAs

[43]	HepG2 cells, palmitate induced insulin resistance	0.1–10 *μ*M for 24 hours	Culture media	↓: TNF-*α*, IL-6

[44]	RAW 264.7 macrophages treated with LPS	5 *μ*M pretreated for 2 hours before LPS treatment for 6 hours	RAW 264.7 macrophages	↓: IL-1*β*, IL-6, MMP9, COX2, and iNOS mRNAs

[45]	Mouse primary splenocytes treated with or without LPS	0.8–3.3 *μ*M for 48 hours	Culture media	↓: TNF-*α*, IL-6

[46]	NIT-1 pancreatic *β*-cells treated with LPS	1.25–5 *μ*M for 24 hours	Culture media	↓: TNF-*α*, IL-6, and MCP-1

[47]	KM mice, obesity and insulin resistance induced by HFD feeding for 13 weeks	50 or 150 mg/kg/d, p.o. for 2 weeks	Serum	↓: TNF-*α*, IL-6

[48]	Wistar rats, STZ 50 mg/kg, single i.p. injection	100 mg/kg/d, p.o. for 7 weeks	Serum	↓: CRP

[49]	Wistar rats, NAFLD and insulin resistance induced by HFD for 8 weeks	187.5 mg/kg/d, p.o. for 4 weeks	Liver	↓: inflammatory cell infiltration

[44]	db/db mice	5 mg/kg/d, i.p. for 4 weeks	White adipose tissue	↓: TNF-*α*, IL-1*β*, IL-6, MCP-1, iNOS, and COX2 mRNAs

[28]	SD rats, STZ 60 mg/kg, single tail vein injection	200 mg/kg/d, p.o. for 12 weeks	Kidney	↓: ICAM-1, TGF-*β*1 protein expression

[50]	NOD mice	200 mg/kg/d, p.o. for 2 weeks	Supernatant from splenocytes, CD4^+^ T cells from spleen/lymph nodes	↓: TNF-*α*, IL-6, IFN*γ,* and IL-17

[51]	NOD mice	50, 150 and 500 mg/kg/d, p.o. for 14 weeks	Supernatant from splenocytes	↑: IL-10/IL-1*β* and IL-10/IL-6 ratios; ↓: IFN*γ*
			Kidney and liver	↑: IL-10/IL-6 and IL-10/TNF-*α* ratios of mRNA levels

↓: decrease, ↑: increase; BBR: berberine, LPS: lipopolysaccharide, KM mice: Kunming mice, HFD: high fat diet, STZ: streptozotocin, NAFLD: nonalcoholic fatty liver disease, p.o.: per os/oral administration, i.p.: intraperitoneal, TNF-*α*: tumor necrosis factor-*α*, IL: interleukin, CRP: C-reaction protein, HP: haptoglobin, MMP9: matrix metalloprotease 9, COX2: cyclooxygenase-2, iNOS: inducible nitric oxide synthase, MCP-1: monocyte chemoattractant protein 1, ICAM-1: intercellular adhesionmolecule-1, TGF-*β*1: transforming growth factor-*β*1, IFN*γ*: interferon-*γ*.

## Abbreviations

BBR: Cerberine
SIRT1: Sirtuin 1
FOXO: Forkhead box O
NADPH: Nicotinamide adenine dinucleotide phosphate
SOD: Superoxide dismutase
AMPK: AMP-activated protein kinase
PI3K: Phosphatidylinositol 3-kinase
Nrf2: Nuclear factor erythroid-2-related factor-2
UCP2: Uncoupling protein 2
GSH: Glutathione
NQO-1: NADPH quinine oxidoreductase-1
HO-1: Heme oxygenase-1
iNOS: Inducible nitric oxide synthase
COX2: Cyclooxygenase-2
ROS: Reactive oxygen species
MAPK: Mitogen-activated protein kinase
IKK-*β*: I*κ*B kinase-*β*
I*κ*B-*α*: Inhibitory *κ*B-*α*
NF-*κ*B: Nuclear factor-*κ*B
AP-1: Activator protein 1
PPAR*γ*: Peroxisome proliferator-activated receptor *γ*.
